# Feasibility and acceptability of a structured quality by design approach to enhancing the rigor of clinical studies at an academic health center

**DOI:** 10.1017/cts.2021.837

**Published:** 2021-08-13

**Authors:** Hamid Moradi, Margaret Schneider, Elani Streja, Dan Cooper

**Affiliations:** 1 Institute for Clinical and Translational Science, University of California Irvine, School of Medicine, Irvine, CA, USA; 2 Tibor Rubin, VA Medical Center, Long Beach, CA, USA; 3 Division of Nephrology, Hypertension and Kidney Transplantation, University of California Irvine, School of Medicine, Irvine, CA, USA

**Keywords:** Quality by design, critical to quality, clinical trials, patient safety, implementation, trial design

## Abstract

**Introduction:**

Clinical trials are a critical step in the meaningful translation of biomedical discoveries into effective diagnostic and therapeutic interventions. Quality by design (QbD) is a framework for embedding quality into the design, conduct, and monitoring of clinical trials. Here we report the feasibility and acceptability of a process for implementing QbD in clinical research at an academic health center via multidisciplinary design studios aimed at identifying and prioritizing critical to quality (CTQ) factors.

**Methods:**

The Clinical Trial Transformation Initiative’s Principles Document served as a guide to identify and categorize key CTQ factors, defined as elements of a clinical trial that are critical to patient safety and data integrity. Individual trials were reviewed in CTQ design studios (CTQ-DS) and the feasibility and acceptability of this intervention was examined through post-meeting interviews and surveys.

**Results:**

Eight clinical research protocols underwent the QbD evaluation process. The protocols ranged from multicenter randomized clinical trials to nonrandomized investigator-initiated studies. A developmental evaluation informed the iterative refinement of the CTQ-DS process, and post-meeting surveys revealed that CTQ-DS were highly valued by principal investigators (PIs) and resulted in multiple protocol changes.

**Conclusions:**

The present study demonstrated that QbD principles can be implemented to inform the design and conduct of clinical research at an academic health center using multidisciplinary design studios aimed at identifying and prioritizing CTQ elements. This approach was well received by the participants including study PIs. Future research will need to evaluate the effectiveness of this approach in improving the quality of clinical research.

## Introduction

The fundamental goal of translational research is to bridge the divide between preclinical discoveries and clinically relevant therapies and interventions. Meaningful translation can only occur with clinical trials that address salient health challenges, efficiently generate accurate evidence, minimize participant risk, assure inclusion of underrepresented populations, and engage all stakeholders at early phases of protocol development [1,2]. However, over the past several years, the clinical trial enterprise has fallen short in achieving many of these goals, and this has led to a crisis of public trust in biomedical research [3–7]. In addition, lack of quality clinical research has jeopardized implementation of discoveries and innovations made over the past few decades, resulting in lost opportunity and adverse economic and public health outcomes. The importance of quality in clinical research has been further highlighted by the current COVID-19 pandemic. The lack of effective and safe therapies has led to streamlined protocol approval and implementation with hasty execution of clinical trials, which in many cases have overwhelmed existing, already strained institutional mechanisms that are supposed to enhance clinical trial quality and safety. In many instances, the lack of key quality elements in COVID-related research has had a counterproductive therapeutic and diagnostic effect [8].

Over the past few decades, there have been various efforts aimed at creating and disseminating innovative approaches that help embed quality metrics into the design, conduct, and monitoring of clinical trials. These efforts have been led by key stakeholders in the global clinical trial enterprise, including international regulatory agencies (such as the Food and Drug Administration [FDA] and International Council for Harmonization [ICH]), the pharmaceutical and medical device industries, National Institute of Health (NIH), and academic institutions [9–12]. One such effort which has gained traction in the last few years leverages and adapts Quality by Design (QbD) concepts frequently used in the manufacturing sectors (i.e., automotive and pharmaceutical manufacturing) for the purpose of clinical research.

The key principles underlying QbD emanate from the endeavors of pioneering scientists in the mid-1900s (e.g., Joseph Juran and W. Edwards Deming [13]) who created systematic approaches to quality improvement. They proposed that instead of judging quality solely by examining the end product of a process, focus should be placed on identifying and addressing key (critical to quality (CTQ)) elements across the whole manufacturing process from start to finish. Furthermore, they expanded the scope of quality improvement beyond sole inspection of process components to include the human dimensions of this process by focusing on the need for enlightened management, constant learning, and mitigating resistance to change [14–16]. While these concepts have been broadly applied in other industries, their application to clinical research has been lagging.

The task of adapting QbD principles to clinical research was initiated by the Clinical Trial Transformation Initiative as part of a multistakeholder project. Significant progress has been made thus far in defining the applicable principles and components of QbD that may be relevant to clinical trials. In this regard, the QbD approach has focused on identifying what is CTQ in a particular study with subsequent tailoring of the protocol design to eliminate unnecessary complexity, avoid predictable errors, as well as devise a focused, efficient, and streamlined monitoring and auditing plan for oversight [17]. CTQ in this context is defined as elements of a study that are key to integrity of data and/or participant safety.

To help stakeholders in clinical research better understand and apply these concepts, Clinical Trial Transformation Initiative has developed a set of CTQ factors, as documented in the CTQ Factors Principles Document, where application of QbD, and specifically systematic, high-quality study planning, has been conceptually described [18]. The CTQ principles were developed collaboratively via a series of multistakeholder workshops involving over 200 attendees with a range of perspectives (including patient advocates, institutional review boards, academic trialists, clinical investigators, clinical research organizations, pharmaceutical and medical device companies, regulatory reviewers, and inspectors) [19,20]. In addition, Clinical Trial Transformation Initiative has developed a suite of resources to support researchers in applying this approach to clinical trials [21]. However, while clinical research-related QbD principles are well described and broadly disseminated, pragmatic illustrations of how these concepts can be put into practice and formally adopted are scarce. This is especially true at academic health centers where research tends to be less centralized. In this manuscript, we describe a formalized pathway for implementing the QbD approach in the design and conduct of clinical research directed at our institution using CTQ design studios (CTQ-DS) and the resources made available at our Clinical and Translational Sciences Award (CTSA) hub.

## Methods

### Institutional Engagement

As the first step in this process, key stakeholders involved in clinical research at UC Irvine were made aware of QbD concepts including CTQ factors. This was accomplished through a one-day workshop sponsored by our CTSA hub (Institute for Clinical and Translational Science [ICTS]) and Clinical Trial Transformation Initiative. The attendees included organizational leaders in the School of Medicine, as well as key personnel involved in clinical research development and administration at our institution. The workshop not only provided a didactic session describing QbD concepts and key components of the CTQ Principles Document but it also involved case studies of previous and prospective trials at UC Irvine in a participatory demonstration of the QbD approach.

This informative session was conducive to educating the UC Irvine clinical research community about the QbD approach and providing practical examples on how this methodology can be used to improve the design and overall quality of clinical research at our institution. Post-workshop surveys confirmed that the majority of attendees favored adoption of QbD concepts in their own research as well as at the institutional level (Table 1). We also discussed potential pathways for implementation of QbD at UC Irvine given that a formal adoption platform is lacking at this time. It was determined that application of QbD concepts would be most appropriate and feasible at early stages of protocol development and study design when there would be an optimal opportunity to address CTQ elements in the design of the study.

**Table 1.** Individual learning objective and statement ratings from workshop attendees (19 of 27 participants responded to the survey)

Learning objectives and statements	% Disagree^1^	Neutral	% Agree^2^
After today’s workshop, I feel that I have a good basic understanding of the QbD principles	0	5	95
I can imagine a pathway whereby QbD principles would be applied widely at UC Irvine	0	5	95
I am interested in applying QbD processes to my own research	0	6	94
I would like to learn more about QbD	6	6	88
I enjoyed the workshop	5	11	84
The case study format was very effective	5	0	95
The content was relevant to me	0	11	89
The content was organized well and easy to follow	0	11	89
I intend to use the strategies I learned in this workshop	6	6	88

QbD, Quality by Design; UC, Irvine-University of California, Irvine.

1 Combination of strongly disagree and somewhat disagree.

2 Combination of strongly agree and somewhat agree.

A smaller planning group subsequently outlined a strategy to construct a core working group with expertise in principles of QbD and various aspects of clinical trial design and invite PIs in the planning stages of clinical research to submit their draft protocol to the working group for feedback. In addition to the core working group, each CTQ-DS would include a member representing the patient perspective as well as content experts specific to the study topic. Rather than being a regulatory hurdle, the CTQ-DS would serve as a resource to investigators and help them design and establish a monitoring plan tailored for their study.

### QbD Working Group

The QbD working group was created with the goal of conducting CTQ-DS for individual clinical trials at the earliest stages of study development. The working group was made up of core members who were selected based on their expertise in areas of critical importance shared by all clinical trials (Table 2), including experts on clinical trial informatics, statistical design, recruitment and retention, and research ethics. The core group also included experienced study coordinators, research nurses, and seasoned clinical trial investigators who provided practical guidance on trial design and conduct. There was also an ad hoc group that included the PI and their study team as well as content experts who were tailored to the needs of the study being discussed and included investigators with scientific expertise (including preclinical experts) in the particular field of study.

**Table 2.** CTQ-DS composition

*Core member expertise*
Moderator
Research ethics
Biostatistics
Clinical trialists and clinical research coordinators/nurses
Informatics
Recruitment and retention
Meeting evaluator
Dissemination and implementation
*Ad hoc attendees*
PI and clinical coordinators of the study under review
Participant advocate
Content expert(s)
Learners (K or T awardees)

CTQ-DS, critical to quality-design studios; PI, principal investigator.

To incorporate the patient perspective into the design and conduct of the study, the ad hoc group also included a patient/participant representative who was selected based on the condition being studied. Awardees of training grants (such as K and T awardees) were also invited to CTQ-DSs where they had a chance to learn and apply QbD concepts in order to further disseminate the QbD approach [22]. Core and ad hoc members of the working group were provided with training on the principles of QbD, goals of the CTQ-DS sessions and their role as members of the design studios in evaluation and prioritization of CTQs. In addition, all participants, both study PIs and CTQ-DS members, were aware that this was a formative research project, and that the intention was to examine the feasibility and acceptability of the CTQ-DS

### CTQ Design Studios

**Fig. 1.**
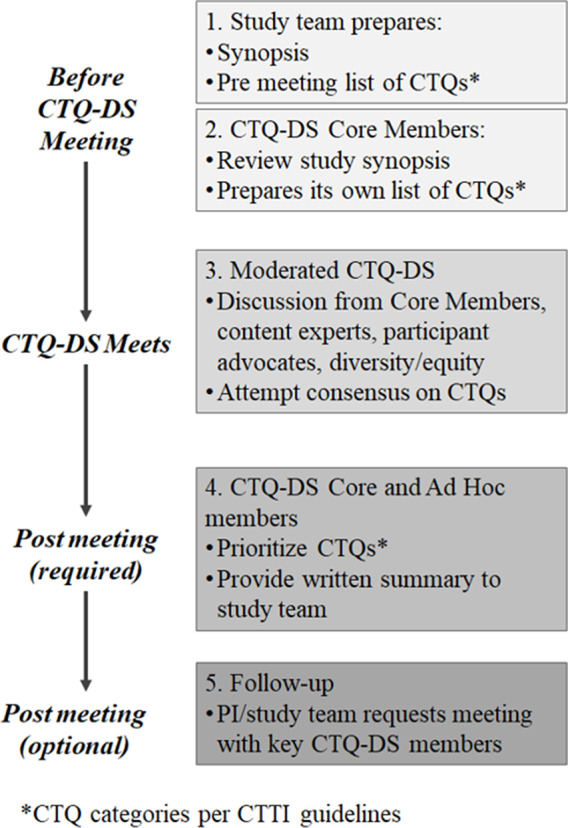
CTQ-DS, critical to quality-design studios; CTQ, critical to quality.

To enable reproducibility and facilitate implementation, the design studios were planned and conducted using a set structure which was regularly assessed and updated (Fig. 1). The process of scheduling a CTQ-DS began with the QbD adoption project leads identifying studies expected to benefit from participation in the design studios (studies in the design stage or those requiring site feasibility and monitoring assessment). The PI responsible for the study was then provided with an overview of the QbD program and invited to participate in the design studios.

Once the PI agreed to partake in this process, a CTQ-DS meeting was scheduled based on the availability of all the members including the patient/participant advocate. The PI provided a 3–4-page description of study rationale, design, and implementation plan (Study Synopsis, Supplement Table 1) one week prior to the meeting in order for the participants to identify a list of preliminary CTQs which were collated in advance of the meeting. On the day of the CTQ-DS, the meeting began with a brief (10-minute) presentation on the main aspects of the trial design by the PI. The session was then opened to discussion including questions and feedback aimed at further clarifying key design, conduct, and monitoring elements of the study. At this time, CTQ elements were identified and shared with the PI. The meeting was conducted as such to ensure feedback was received from all participants of the design studio, especially the patient/participant advocate.

In addition to sessions being recorded, a notetaker documented all elements of the discussion at the time of the meeting. Post-meeting, a list of CTQ factors was compiled based on the notes and recording from the session, and according to the different categories of CTQs codified in the Principle’s Document. Final prioritization of the CTQs was accomplished using a REDCap survey where the CTQ-DS attendees were asked to rate the priority score of each CTQ [23]. REDCap (Research Electronic Data Capture) is a secure, web-based software platform designed to support data capture for research studies, providing 1) an intuitive interface for validated data capture; 2) audit trails for tracking data manipulation and export procedures; 3) automated export procedures for seamless data downloads to common statistical packages; and 4) procedures for data integration and interoperability with external sources. The priority scoring system was evaluated and optimized continuously based on the feedback from the attendees (see Supplement Table 2 for definitions). Once a prioritized list of CTQs was compiled, a design studio report was generated using a set template that has been developed over the course of this project. The report began by providing a brief background on QbD concepts and the purpose of the CTQ-DS. Subsequently, descriptions regarding the meaning of the report, its findings, and helpful references were provided. Finally, tables listing CTQs along with their priority scores, beginning with a table of top 10–15 CTQs (those with priority scores in the one range) followed by tables of other CTQs organized based on their CTQ category, were provided in the report. The report was then emailed to the PI and research team. The details involved in selection of a study, planning, and conducting CTQ-DS were used to create a standard operating procedure (SOP) (Supplement Figure 1). Surveys of the participants and PIs were used to improve the procedures and modify the SOP as well as the CTQ prioritization scheme.

### Developmental Evaluation

Using a developmental approach to evaluation [24], the form of data collection utilized to evaluate working group members’ perceptions of the process evolved over time and informed implementation of the CTQ-DS iteratively. Following each of the first two CTQ-DS, a REDCap survey of all participants assessed their perceptions of the meeting format, composition of the working group, and meeting efficacy. In addition, a follow-up interview elicited suggestions for improving the process. Beginning with the second meeting, an approach to prioritizing CTQs was introduced on the post-meeting REDCap survey, and an open-ended item at the end of the survey asked for feedback on the prioritization approach. Based on this feedback, a revised prioritization process was implemented for the last four meetings, with an open-ended survey item retained to facilitate ongoing feedback.

Perceived impact of the CTQ-DS from the perspective of all participating PIs was assessed with a REDCap survey administered in April of 2021. Thus, the time elapsed between the CTQ-DS and the follow-up PI survey ranged from a minimum of 2 months to a maximum of 20 months. On the follow-up survey, PIs were asked to report on the status of their study, their perceptions of the utility of the CTQ-DS, and what action, if any, they had taken with respect to each of the CTQs identified in the report that they were provided following the CTQ-DS.

## Results

### Overall Description

Over the past 2 years, our team has reviewed eight separate clinical research protocols via the CTQ-DS at UC Irvine, including five during the COVID-19 pandemic. While the initial three meetings were conducted in-person and via roundtable discussions, due to the social distancing restrictions put in place during the pandemic, the remaining five meetings were conducted using virtual technologies. We found that conducting virtual meetings did not decrease participation or enthusiasm for holding CTQ-DS. In fact, by using virtual technologies, we were able to reduce the barriers posed by commute time and geography. The studies that underwent the QbD process via CTQ-DS included a wide range of clinical research designs (Table 3). Half (four) of the studios were devoted to studies focused on diagnostic and therapeutic challenges posed by the COVID-19 pandemic. They included multisite randomized clinical trials which among other areas needed evaluation of potential CTQs related to the feasibility of these studies at our institution. Many of the CTQs identified helped clarify the resources needed to initiate these trials at our site which typically are not addressed in trial protocols created for multicenter trials. For instance, an international COVID-therapy trial was found to require significant resource development and staff training for successful site initiation. Awareness of these issues was relevant to the PI’s implementation plan for our site. There were also smaller investigator-initiated studies that took advantage of the wide range of expertise being represented in the CTQ-DS to help develop more effective study design and management plans. Concentrating on identification and prioritization of CTQ elements helped embed QbD concepts in a focused and organized manner that could be replicated across all studies.

**Table 3.** CTQ design studios conducted at UC Irvine

Study title	Participant advocate	CTQ-related insight (per CTTI category)
Diabetic medication and the gut microbiome	Patient with type 1 diabetes	Feasibility: Recruit in the clinic on the day of the visit to minimize study participation duration
Phase 1/2a study of the safety and tolerability of neurosurgical stem cell transplantation for a rare neurologic disorder	Patient advocate who had lost several family members to the disorder	Study conduct: Construct a detailed plan for explaining the meaning of a placebo-controlled study for patients with the potential of developing the rare disorder
The nutrient trial: nutritional intervention among myeloproliferative neoplasms	Patient with myeloproliferative neoplasms	Patient safety: Need to monitor to ensure that patient is not experiencing a weight maintenance issue
Convalescent plasma in patients with COVID-19 (randomized clinical trial)	Recovered COVID-19 patient who volunteered to donate plasma	Protocol design: Effective messaging for participant recruitment should be developed with input from the target population
The effect of mitigation procedures to inhibit COVID-19 transmission as K-12 schools reopen	Mother and daughter (6th grader) in one of the selected schools	Patient safety: Make every attempt to not identify and possibly embarrass the students who volunteer for the study
Dietary intervention and blood pressure in living kidney donors: single-blind randomized controlled trial	Healthy subject post kidney donation	Protocol design: The eligibility window for recruitment should take into account the post-surgical recovery period, during which patients may be in pain and/or taking pain meds
ACTIV-1 IM: randomized master protocol for immune modulators for treating COVID-19	Patient who had recovered from COVID-19 infection	Feasibility: This study has daily data collection and will take a lot of effort and coordination. Therefore, budget and staffing needs should be assessed and addressed in preparation of study conduct/monitoring
Serosurveillance of UC Irvine health staff for SARS-CoV-2 antibodies during COVID-19 outbreak using a coronavirus antigen microarray	Health staff working at the medical center	Study conduct: At least one member of the DSMB should be from outside UCI to minimize the potential for unconscious bias

COVID19, coronavirus disease 2019; CTQ, critical to quality; CTTI, clinical trial transformation initiative; DSMB, data safety monitoring board; SARS-CoV-2, severe acute respiratory syndrome coronavirus 2; UC Irvine, University of California, Irvine.

Each session included a patient/participant advocate who played a crucial role in bringing the patient perspective to the design and monitoring plan of the study. The feedback from the patient advocate was especially key in addressing the recruitment and retention concerns of the studies reviewed (see Table 4). Factors identified by the patient representatives were mostly relevant to research participant recruitment and retention, which is commonly acknowledged as a challenge to clinical research success [25,26].

**Table 4.** Examples of CTQs identified by patient representatives at each studio

Patient representative recommendation	Performance outcome	Hoped-for outcome
At what point will you tell the patient they received the placebo? These patients have psychiatric issues and pose a suicide risk. You can§t just enroll them and just let them go off thinking they received the drug. You need to tell them the truth, the way things are. They need education and you have to stay with them	Adverse events participant retention	Development of a protocol for revealing group assignment to the participant and supporting mental well-being afterwards
Weight loss or weight gain can be an issue in patients with Myeloproliferative Neoplasms. It will be important to track weight status among the participants and respond in the event that the weight is moving in a direction that is not indicated for that patient	Unintended effects participant retention	Integration of weight monitoring into the assessment plan and development of a protocol for responding to weight changes
Waiting until after the participant has been to see their physician and collected the prescription before you recruit means that you have a high probability of losing the participant. The best plan would be to recruit in the clinic on the day of the visit	Recruitment	Modify the recruitment plan to invite patients into the study on the day of their clinic visit
Effective messaging for participant recruitment should be developed with input from the target population	Recruitment	Recruitment materials communicate eligibility criteria clearly to maximize the probability that interested patients will qualify
It might be helpful if an email from CEO, Director of EIP, or other leadership is sent to encourage response to upcoming recruitment email. This strategy might enhance your penetration into the population for recruitment	Recruitment	Modify the recruitment plan to include an email to health care workers encouraging attention to the recruitment email
Involve participants/parents, teachers, school staff and OCHCA in the development of the informed consent document. Write the consent form so that it is meaningful to the target audience, participants	Recruitment	Elicit input from the community stakeholders prior to finalizing the consent form
From the patient perspective, the first few weeks after donation might not be a time when this study would be appealing. By about 4 months, patients may be feeling sufficiently recovered to be willing to volunteer	Recruitment	Modify timing of recruitment to give patient time to recover from the surgery
Consider providing compensation to participants for all assessments requiring in-person visits, this can help with accrual and retention	Recruitment retention	Add participant compensation
Be clear with potential participants up front about the expectation that they will be asked to refrain from additional changes to their pain medication for 2 months and that it could take up to 2 months for them to experience pain relief even if the medication is working	Recruitment retention	Enhance consenting procedures to ensure participant comprehension of the implications of participation for their medication regimen.

CTQ, critical to quality; CEO, chief executive officer; OCHCA, Orange County health care agency; EIP, epidemiology and infection prevention.

### Developmental Evaluation

Supplement Table 3 depicts how the implementation of the CTQ-DS evolved over time and Supplement Table 4 describes the post-meeting data that informed refinements in the format of the studio and the prioritization task. As shown in Supplement Table 3, the size of the working group declined slightly over time, stabilizing at about a dozen members. Most of the attrition occurred after the first few studios and reflected both some programmatic streamlining of membership to avoid duplication of expertise and some self-selection of continued membership. The first study, which was a stem cell trial involving sham surgery, generated the largest number of CTQs owing to the complexity of the protocol and the many ethical and patient safety issues that arose. The remaining studies generated an average of 30 CTQs each (range = 24–38), suggesting that the stem cell trial was an outlier. The post-meeting data collection (Supplement Table 4) led to improvements in the way that pre-meeting materials were formatted and delivered to the working group as well as how the meeting itself was structured. Similarly, feedback on the prioritization scoring method led to implementation of a simplified version for the last four studios, with indications that this approach was highly acceptable to working group members.

All eight PIs who participated in the CTQ-DS responded to an online survey asking about their experience (Table 5). Responses indicated overwhelming enthusiasm for the program. All of the PIs found the experience useful and would take advantage of it again if offered the opportunity. Moreover, all of the PIs reported having taken action or intending to take action on the majority of the CTQs that emerged from the studio except for one study (Study #8), which was a multicenter international trial for which the protocol was already fixed and addressing CTQs in the design and monitoring plan was not feasible. Omitting this multicenter trial, PIs reported on average that they modified their study protocol to address 59% of the CTQ factors identified in their report. In comments entered on the survey, PIs reported specific benefits such as, “There were a number of things I had not yet begun to think about with respect to clinical needs” and “We modified our approach to both data collection and oversight/data integrity based on the feedback.” In particular, the report that the PIs received was noted as helpful, with one respondent indicating that “It was incredibly thoughtful and thorough and elements of it were actually very useful to transcribe almost directly into subsequent grant applications,” and another stating that the report “Gave me discrete items to address for improvements.” All of the PIs reported that the studies had moved forward since the CTQ-DS with concrete progress reported in terms of opening the study for accrual, setting up clinical infrastructure, revising the proposal and submitting to the IRB, submitting a proposal to the NIH, and/or accruing samples for analysis.

**Table 5.** Results of the follow-Up PI survey

	CTQ studio							
	1	2	3	4	5	6	7	8
Time elapsed in weeks from studio to survey	94	78	63	50	47	42	26	13
How useful was the CTQ-DS? 1. not at all; 2. a little; 3. pretty; 4. extremely	4	4	3	3	4	4	4	4
How satisfied were you with the report? 1. not at all; 2. a little; 3. pretty; 4. extremely	4	4	4	4	4	4	4	4
How likely would you be to do it again? 1. not at all; 2. a little; 3. pretty; 4. extremely	4	4	3	4	4	4	4	4
Percent of CTQs used to modify protocol	17	55	36	100	72	51	83	0
Percent of CTQs used to modify plans	44	13	44	0	2	40	16	0
Percent of CTQs with intention to address	32	24	16	0	8	7	2	0
Percent of CTQs with no action taken	5	11	8	0	0	0	0	100

CTQ-DS, critical to quality-design studios; CTQ, critical to quality, PI, principal investigator.

## Discussion

The principles of QbD have been developed and adopted in various industries, including in pharmaceutical manufacturing. More recently, their potential application in clinical research has been described through multistakeholder efforts, which have included regulators, members of academia, as well as the pharmaceutical and medical device industry. While there are many resources available that describe QbD concepts as they relate to biomedical research, specific examples for adoption and implementation of this approach have remained lacking. In this manuscript, we have described a formal pathway for implementation of QbD at an academic health center using identification and prioritization of CTQ factors, which are key components of this approach. We achieved this through establishment of a formal CTQ-DS with the goal of embedding QbD principles into the design and management plan of clinical research protocols proposed at our institution. During the past 2 years, we conducted eight CTQ-DS, each reviewing a unique clinical research study. The majority of the meetings were held virtually due to COVID-19 pandemic restrictions.

We continuously improved the planning, conduct, and reporting process for our design studios and based on the latter knowledge, we devised a standard operating procedure that can be adapted for settings outside of our institution. Finally, we conducted a survey to evaluate the participating PIs’ perception of value and the utility of CTQ-DS in improving the design and conduct of their study. The results show that CTQ-DS were well received and have played a valuable role in helping PIs proactively anticipate aspects of the trial protocol that were deemed to be important and adjust their protocol accordingly to address these findings. The only study that did not report protocol changes in response to the CTQ-DS was a multisite clinical trial. This finding is not surprising given the many difficulties involved in changing the design of studies that have already been finalized, especially multicenter studies, which have already gone through many iterations and are less amenable to change. Therefore, our findings indicate that CTQ-DS are most useful for clinical studies at the design stage. However, it is possible that even for a finalized protocol the findings from a CTQ-DS could still be helpful in creating a site-specific trial management plan and improving the feasibility of the study at a single implementing institution, although this remains to be determined in future follow-up evaluations.

There are several important aspects of our efforts that merit highlighting. In recent years, there has been increasing recognition of the critical need for a team-science approach to the development and conduct of translational research [27–30]. This approach promotes engagement of a multidisciplinary, multistakeholder team in the design, conduct, and dissemination of clinical research in order to generate knowledge that is impactful and of relevance not only to the scientific community (researchers), but also to the society as a whole. Our QbD implementation methodology has been uniquely suited to facilitate team science and stakeholder engagement. In our CTQ-DS, we included members with a wide range of expertise and skillsets to help identify the diverse CTQs unique to each study. The team science approach was manifested not only by including multidisciplinary members in our core team but also through involvement of ad hoc content experts, community representatives, and members. Additionally, the formative feedback from CTQ-DS which was communicated in a collegial and helpful manner created an environment of collaboration rather than evaluation. Therefore, the design studios were offered as a resource to help better design and manage clinical studies by the research teams rather than a regulatory hurdle. In this feasibility study, PIs had the option to include members of their study team in the studio, but few availed themselves of this opportunity. A case could be made that including study team members would increase the likelihood that CTQs would be successfully addressed in the protocol. Future evaluation of the studios should incorporate this variable in the evaluation design

The role played by participant advocates in the design of clinical trials is also critical. Novel approaches, such as the use of community engagement studios [31], are increasingly embraced by CTSA hubs in an effort to embed the unique voice of patients and prospective volunteers in early phases of trial design. Clinical Trial Transformation Initiative has also emphasized the beneficial role played by patient and community advocates in QbD [32]. We found that inclusion of patient/participant advocates in our design studios was an effective method for incorporating the perspective of study subjects into the design and implementation plan of clinical studies. In addition, their input played a key role in several aspects of study design, most notably in the recruitment and retention plan as well as selection of patient-relevant endpoints.

The implementation of QbD via CTQ-DS not only was well received by our PIs as a resource to help them better design and develop their studies but it also provided an opportunity for our researchers to incorporate the latest recommendations of the upcoming ICH E8 and ICH E6 renovations into their clinical research protocols [33,34]. These internationally recognized guidance documents, which cover good clinical practices for clinical trials, now include a section on the consideration of quality in the design and conduct of clinical studies. Furthermore, special emphasis has been placed on identification and management of CTQ factors. Therefore, adoption of QbD principles through implementation of CTQ-DS has helped our team stay at the forefront of these efforts aimed at incorporating quality into clinical research design and development. Quality in this context is defined by Clinical Trial Transformation Initiative as “lack of errors that matter,” such as errors that compromise patient/participant safety or the integrity of the data and results of the study. Future work extending the current findings will evaluate whether the CTQ-DS is effective for preventing errors that matter in the conduct of clinical research being carried out at an academic health center.

Some of the limitations of our approach also need to be mentioned. Our system for applying QbD concepts is especially suited for academic institutions and adoption of a similar scheme in smaller organizations may be challenging. At academic health centers, we have access to a wide pool of experts to populate our working group. Moreover, these individuals were willing to participate as an element of their University Service and did not require compensation. This approach may not be feasible in smaller organizations or those with differing priorities. However, we believe that the concepts highlighted in our study (such as team science, community engagement, and design studios) can be adapted and tailored for other settings, including commercial settings. For instance, partnerships can be formed between multiple organizations with the goal of creating CTQ-DS that can help review a variety of external and internal studies, much like central and commercial institutional review boards. Another important limitation of the QbD approach is that it remains to be demonstrated that adoption of QbD or any other mechanism for incorporating quality metrics into clinical research will in fact improve quality of clinical studies. Therefore, it is important that future, carefully designed and controlled studies address this critical question (whether adoption of QbD or other quality assurance methodologies in fact improves quality of clinical research). In addition, relevant qualitative and quantitative endpoints need to be identified and validated in order to help with evaluation of QbD efficacy in improving quality of clinical research. In fact, we are planning studies to address these important gaps in order to plan future larger studies examining QbD effectiveness. Nevertheless, a formalized approach for implementation of QbD at academic health centers, which can then be adopted at other institutions, will be the critical first step to enable future research to test the comparative effectiveness of QbD (as opposed to current common practices) in improving quality in clinical research. Hence, our approach for operationalizing QbD can serve as a platform for adoption of QbD at other academic health centers and thereby facilitate future multicenter effectiveness evaluation of the QbD approach. Finally, there are pitfalls that we encountered during the implementation of our approach that other investigators need to be aware of and account for in their planning. First, CTQ-DS require participation of many faculty, staff, and other participants and this requires time commitment and coordination of schedules for a sizeable number of individuals. Sessions need to be planned and scheduled 1–2 months in advance in order to facilitate this process. Second, identification of clinical studies and PIs who are eligible and willing to participate in design studios requires planning and support from organizational areas involved in clinical research (Center for Clinical Research, Institutional Review Boards, etc). We found that this process needs to be planned several months in advance in order to help successful identification of a project and to assist PIs with some of the requirements of this process (i.e. Study synopsis generation, creation of a study presentation, education regarding the QbD process and design studios). Preplanning is also critical to include patient representatives in the design studios. Most of our patient representatives were identified and invited by the PI of the under review, but in cases where these PIs lacked such contacts, it was useful to reach out to the Community Engagement Unit of our Clinical and Translational Science Award hub for assistance. Lastly, ongoing surveys and feedback from participants have been used to optimize many of the operational aspects of the design studios. These included the CTQ identification a scheme and the scoring system used to prioritize CTQs. Therefore, continuous evaluation and improvement of the CTQ-DS processes are crucial to the successful implementation of this approach in a changing academic environment.

In conclusion, we have demonstrated that adoption of QbD concepts is feasible through administration of multidisciplinary design studios focused on CTQ factors specific to each study. Moreover, the studios were perceived as extremely valuable by the participating PIs and stimulated PIs to address the identified factors in their protocol revisions. Future studies will need to determine whether implementation of QbD using this approach improves quality of clinical research at academic health centers.

## Supplementary material

For supplementary material accompanying this paper visit http://dx.doi.org/10.1017/cts.2021.837. click here to view supplementary material
