# Supplementary material for: Feasibility and acceptability of a structured quality by design approach to enhancing the rigor of clinical studies at an academic health center
Source: J Clin Transl Sci. 2021 Aug 13;5(1):e175. doi: 10.1017/cts.2021.837 (PMC8596060; doi:10.1017/cts.2021.837)
Supplement: Supplementary file 1 [file S2059866121008372sup001.docx]

**Supplementary Material**

**Supplement Table 1. Topics Covered in the Brief Study Synopsis**

| Title | Brief rationale | Planned enrollment | Treatment allocation |
| --- | --- | --- | --- |
| Primary objectives | Secondary objectives | Inclusion criteria | Exclusion criteria |
| Randomization | Assessments | Study duration | Withdrawal |
| Follow-up | Data monitoring | Statistical methods |  |

**Supplement Table 2. CTQ Priority Score Definitions**

| *High Priority* | This factor is critical to the integrity of the research. If the investigators do not attend to this concern, the study is at high risk for failure, either in terms of an inability to execute the protocol (e.g., recruit adequate participants) or in terms of a design flaw that will greatly reduce confidence in the findings. |
| --- | --- |
| *Medium Priority* | This factor has the potential to threaten the feasibility of, and/or confidence in, the study. The quality of the data and/or the strength of the results are highly likely to be meaningfully improved by attending to this factor. |
| *Low Priority* | This factor should be acknowledged as a potential threat to study integrity. If resources or pragmatic concerns prevent the investigators from addressing this concern, the study should still proceed, but the investigators should anticipate and plan for challenges related to this factor. |

Abbreviations:, CTQ-critical to quality

| **Supplement Table 3. Overall Structure of 8 CTQ-DS** | | | |
| --- | --- | --- | --- |
| **CTQ-DS** | **No. Working Group Members** | **No. CTQs** | **Prioritization Instructions** |
| 1 | 19 | 70 | None |
| 2 | 16 | 24 | Imagine you have $100 to allocate across all of the different recommendations listed below. You should allocate more money to the recommendations that you feel have the highest priority. You can allocate any amount from $0 to $100 to each item, but in the end the total must add up to $100. |
| 3 | 14 | 25 | Same as above |
| 4 | 16 | 31 | Same as above |
| 5 | 13 | 25 | Please prioritize the CTQ factors by  designating each CTQ as High, Medium, or Low priority ( Supplement Table 2). |
| 6 | 12 | 37 | Same as above |
| 7 | 12 | 38 | Same as above |
| 8 | 11 | 30 | Same as above |

Abbreviations: CTQ-DS-critical to quality-design studios, CTQ-critical to quality, No.-number of

| **Supplement Table 4. Developmental Evaluation and Ongoing Refinement of CTQ-DS** | | |
| --- | --- | --- |
| **CTQ-DS** | **Post-Meeting Evaluation Results** | **Feedback** |
| 1 | “Meeting was a good use of my time”: 95%  “Meeting generated useful information for the investigator”: 100%  “Would be willing to serve as a standing member”: 89% | *Preparation*: All materials should be provided ahead of time in consolidated email.  *Structure*: Invite discussion according to QbD principles rather that going around the room. |
| 2 | “Meeting was a good use of my time”: 100%  “Meeting generated useful information for the investigator”: 100%  “Would be willing to serve as a standing member”: 100% | *Preparation*: Encourage working group members to submit top 3 CTQs ahead of time.  *Structure*: Impose a strict time limit on the PI presentation. |
| 3 | Open-ended survey question only | *Prioritization*: Too complicated. |
| 4 | Open-ended survey question only | *Prioritization*: A pain |
| 5 | Open-ended survey question only | *Prioritization*: Much quicker and simpler to review. Thank you! |
| 6 | Open-ended survey question only | *Prioritization*: This is a good way of prioritizing CTQ factors. |
| 7 | Open-ended survey question only | *Prioritization*: This technique for prioritizing Critical Quality factors seems good to me. |
| 8 | Open-ended survey question only | *Prioritization*: It is quicker, but doesn't allow for any comments - if they might help provide info about chosen priority. |

Abbreviations: CTQ-DS-critical to quality-design studios, CTQ-critical to quality, PI-principal investigator

Step 1. Identifying an appropriate time and date for the meeting.

- *Meetings are held on a quarterly basis and are scheduled in advance based on availability of core members.*
- *These include: expert in statistics, bioinformatics, recruitment, ethics, diversity, clinical trial experts, experienced research nurse and study coordinator, evaluation expert, note-taker and ICTS leadership*

Step 2. Study of interest is identified and PI invited for participation in the QbD adoption project (CTQ-DS).

- *This step is completed as a collaborative effort between QbD project lead and ICTS leadership*
- *The PI emailed a brief description of QbD using a standard format (email template available)*

Step 3. Once study PI has expressed interest and availability for the scheduled CTQ-DS meeting.

- *Subject matter experts and patient/patient advocates are identified, invited and confirm attendance*
- *Brief synopsis provided by the PI to be received by ICTS 4-5 days (preferably over the weekend before) in advance of the scheduled meeting (template file available)*

Step 4. Attendees use the Reviewer Assessment Sheet (see appendix) to enumerate their CTQs prior to the meeting.

- *This sheet includes the 6 categories of CTQs as outlined by CTTI Principles Document*
- *It is used to organize CTQs and provide preliminary comments from each attendee to be completed and emailed prior to the CTQ-DS meeting*
- *Master list of all CTQs which is color coded for each author is emailed to the project lead*
- *These CTQs are used as a platform to guide preliminary discussions during CTQ-DS*

Step 5. CTQ-DS meeting:

- *Meeting duration limited to 2 hours*
- *Brief introduction provided by QbD project lead*
  1. *Meeting agenda*
  2. *Introduction of members of the design studio, PI and research team and the study*
- *Study PI provides a 10-minute presentation on background, hypothesis, design and all necessary elements of the trial*
- *Attendees ask questions and discuss CTQs already identified and new CTQs*
- *Last 10 minutes used for closing remarks and finalization of CTQs*
- *Meetings are recorded, and detailed notes taken*

Step 6. Post CTQ-DS meeting.

- *All CTQs discussed are compiled into a single document*
- *REDCap survey generated and emailed to CTQ-DS members which allows for prioritization of CTQs based on a 1-3 value system (1 high priority, 2 medium and 3 low priority)*
- *Based on the survey results, a report is generated and emailed to the PI which includes a prioritized list of all CTQs and other meeting notes (the report also provides a brief description of QbD and the findings of the CTQ-DS)*
- *The report also contains a table with the top 10 CTQs*
- *PI may request a second meeting to further discuss study after addressing CTQs*

**Supplement Figure 1. Standard operating procedure for CTQ-DS meetings**

Step 7. Incorporation of CTQs into trial design and monitoring

- *A second meeting is offered to be held with the PI and research team as well as the CTQ-DS group where the PI can discuss how each CTQ was incorporated into trial design and monitoring plan.*
- *REDCap survey generated and emailed to CTQ-DS members which allows for prioritization of CTQs based on a 1-3 value system (1 high priority, 2 medium and 3 low priority)*
- *Based on the survey results, a report is generated and emailed to the PI (template report available) which includes a prioritized list of all CTQs and other meeting notes*

Abbreviations: CTQ-DS-critical to quality-design studios, CTQ-critical to quality, PI-principal investigator, ICTS-Institute for Clinical and Translational Science, REDCap- Research Electronic Data Capture
